# Bildgebung nach Unfall in Klinik und Praxis bei Kindern und Jugendlichen: Teil 1 der Ergebnisse einer bundesweiten Online-Umfrage der Sektion Kindertraumatologie der Deutschen Gesellschaft für Unfallchirurgie

**DOI:** 10.1007/s00113-021-01115-2

**Published:** 2021-12-16

**Authors:** Klaus Dresing, Ralf Kraus, Francisco Fernandez, Peter Schmittenbecher, Kaya Dresing, Peter Strohm, Christopher Spering, Sektion Kindertraumatologie der Deutschen Gesellschaft für Unfallchirurgie

**Affiliations:** 1 Klinik für Unfallchirurgie, Orthopädie und Plastische Chirurgie, Universitätsmedizin Göttingen, Robert-Koch-Str. 40, 37099 Göttingen, Deutschland; 2 Klinik für Unfallchirurgie und Orthopädie, Klinikum Bad Hersfeld, Bad Hersfeld, Deutschland; 3 Kindertraumatologie, Klinikum Stuttgart Olgahospital, Stuttgart, Deutschland; 4 Kinderchirurgische Klinik, Städtisches Klinikum Karlsruhe, Karlsruhe, Deutschland; 5 Darmstädter Kinderkliniken Prinzessin Margaret, Darmstadt, Deutschland; 6 Klinik für Orthopädie und Unfallchirurgie, Klinikum Bamberg, Bamberg, Deutschland; 7 Sektion Kindertraumatologie der Deutschen Gesellschaft für Unfallchirurgie, Berlin, Deutschland

**Keywords:** Röntgendiagnostik nach Unfall, Kindesalter, Jugendalter, Strahlenschutz, Deutschlandweite Umfrage, X‑ray diagnostics after trauma, Childhood, Adolescence, Radiation protection, Germany-wide survey

## Abstract

**Hintergrund:**

Die Indikation zum Röntgen sollte bei pädiatrischen und jugendlichen Traumapatienten streng dem ALARA-Prinzip (as low as reasonable achievable) folgen. Die Wirkung der Strahlung auf das wachsende sensible Gewebe dieser Patienten darf nicht außer acht gelassen werden.

**Fragestellung:**

Die Sektion Kindertraumatologie der Deutschen Gesellschaft für Unfallchirurgie (SKT) wollte klären wie in der Traumaversorgung dem Prinzip gefolgt wird.

**Methoden:**

Eine Online-Umfrage war 10 Wochen lang offen. Zielgruppen waren Unfall-, Kinder- und Allgemeinchirurgen sowie Orthopäden.

**Ergebnisse:**

Von 15.11.2019 bis 29.02.2020 beteiligten sich 788 Ärzte: Niederlassung 20,56 %, MVZ 4,31 %, Krankenhaus 75,13 %; Assistenzarzt 16,62 %, Oberarzt 38,07 %, Chefarzt 22,59 %. Nach Facharztqualifikation ergab sich die Verteilung: 38,34 % Chirurgie, 33,16 % Unfallchirurgie, 36,66 % spezielle Unfallchirurgie, 70,34 % Orthopädie und Unfallchirurgie, 18,78 % Kinderchirurgie. Häufigkeit des Kontakts mit Frakturen in der o. g. Altersgruppe wurde angegeben mit 37 % < 10/Monat, 27 % < 20/M, 36 %> 20/M. Etwa 52 % fordern immer Röntgenaufnahmen in 2 Ebenen nach akutem Trauma. Das Röntgen der Gegenseite bei unklaren Befunden lehnen 70 % ab. 23 % wenden die Sonographie regelmäßig in der Frakturdiagnostik an. Bei polytraumatisierten Kindern und Jugendlichen wird das Ganzkörper-CT bei 18 % nie, bei 50 % selten und bei 14 % standardmäßig eingesetzt.

**Diskussion:**

Die Analyse zeigt, dass es kein einheitliches radiologisches Management von Kindern und Jugendlichen mit Frakturen unter den Befragten gibt.

**Schlussfolgerung:**

Vergleicht man die Ergebnisse der Umfrage mit den kürzlich in dieser Zeitschrift veröffentlichten Konsensergebnissen des SKT, so bedarf es noch Überzeugungsarbeit, um den Einsatz von Röntgenstrahlen bei der Primärdiagnostik zu ändern.

## Abstract (en)

**Background:**

The indication for radiography should strictly follow the ALARA (as low as reasonably achievable) principle in pediatric and adolescent trauma patients. The effect of radiation on the growing sensitive tissue of these patients should not be disregarded.

**Question:**

The Pediatric Traumatology Section (SKT) of the German Trauma Society (DGU) wanted to clarify how the principle is followed in trauma care.

**Methods:**

An online survey was open for 10 weeks. Target groups were trauma surgeons, pediatric surgeons, general surgeons, and orthopedic surgeons.

**Results:**

From Nov. 15, 2019, to Feb. 29, 2020, 788 physicians participated: branch office 20.56%, MVZ 4.31%, hospital 75.13%; resident 16.62%, senior 38.07%, chief 22.59%. By specialist qualification, the distribution was: 38.34% surgery, 33.16% trauma surgery, 36.66% special trauma surgery, 70.34% orthopedics and trauma surgery, 18.78% pediatric surgery. Frequency of contact with fractures in the above age group was reported as 37% < 10/month, 27% < 20/M, 36% > 20/M. About 52% always request radiographs in 2 planes after acute trauma. X-ray of the opposite side for unclear findings was rejected by 70%. 23% use sonography regularly in fracture diagnosis. In polytrauma children and adolescents, whole-body CT is never used in 18%, rarely in 50%, and standard in 14%.

**Discussion:**

The analysis shows that there is no uniform radiological management of children and adolescents with fractures among the respondents.

**Conclusion:**

Comparing the results of the survey with the consensus findings of the SKT recently published in this journal, persuasion is still needed to change the use of radiography in primary diagnosis.

## Hintergrund

Der Einsatz der Röntgendiagnostik bei Frakturen ist seit Jahrzehnten auch bei Verletzten im Kindes- und Jugendalter Standard. Den Vorteilen dieser Röntgendiagnostik stehen die Nachteile durch potenzielle Strahlenschäden gegenüber.

Die Sektion Kindertraumatologie (SKT) der DGU (Deutsche Gesellschaft für Unfallchirurgie) wollte mit Teil 1 dieser Umfrage den Status quo in der Diagnostik von knöchernen Verletzungen und Mehrfachverletzungen im Kindes- und Jugendalter erfragen. Es sollte die Frage beantwortet werden, wie die in der Kindertraumatologie tätigen Ärzte die Indikation zur primären Diagnostik sehen? Ziel der Untersuchung war es, die aktuelle Situation des Einsatzes der Bildgebung bei akuten Verletzungen von Kindern und Jugendlichen in Deutschland abzuklären.

## Methodik

Mit dem Programm SurveyMonkey (momentive ai, Europe UC, Dublin, Irland) [59] wurde die Umfrage über den Einsatz der Röntgenstrahlen bei Kindern und Jugendlichen über das Portal der Deutschen Gesellschaft für Orthopädie und Unfallchirurgie (DGOU) durchgeführt. Zusätzlich wurden über den E‑Mail-Verteiler der Fachgesellschaft Kolleginnen und Kollegen aus Unfallchirurgie, Unfallchirurgie und Orthopädie, Orthopädie, Kinderchirurgie und Kinderorthopädie auf die Umfrage über den Einsatz der Röntgenstrahlen bei Kindern und Jugendlichen hingewiesen. Als Instrument wurde SurveyMonkey benutzt. Die Umfrage war freiwillig und erfolgte anonym.

Neben epidemiologischen Fragen wie Tätigkeitsprofilen, Weiterbildungen wurden Fragen zur Häufigkeit der Versorgung und primären Diagnostik von Frakturen und Verletzungen in der Altersgruppe gestellt.

Die Auswertfunktion des Umfrageprogramms lieferte anonymisierte Daten im Excelformat. Die weitere Datenanalyse erfolgte mit dem Datenbankprogramm Filemaker [33] und mit JASP [39]. Ermittelt wurden Daten der zentralen Tendenz und prozentuale Verteilungen.

## Ergebnisse

### Tätigkeit in Klinik und Praxis^1^

An der Umfrage vom 15.11.2019 bis zum 29.02.2020 nahmen 788 Personen teil. 20,56 % (162) waren in der Niederlassung in eigener Praxis, 4,31 % (34) im MVZ und 75,13 % (592) im Krankenhaus tätig; 16,62 % (131) als Assistenzarzt in der Weiterbildung, 38,07 % (300) als Oberarzt und 22,59 % (178) in der Position als Chefarzt beschäftigt.

### Weiterbildung

Bei Fragen zur Weiterbildung wurden sämtliche Weiterbildungen abgefragt, sodass sich durch Mehrfachnennungen Überschneidungen ergeben. Für alle Teilnehmer der Umfrage zeigen sich folgende Facharztqualifikationen: 38,34 % Chirurgie, 33,16 % Unfallchirurgie, 36,66 % spezielle Unfallchirurgie, 70,34 % Orthopädie und Unfallchirurgie sowie 18,78 % Kinderchirurgie. Bei den Assistenten und den Oberärzten zeigt sich entsprechend der neuen Weiterbildungsordnung der überwiegende Anteil im Fach Orthopädie und Unfallchirurgie, Tab. 1. Der Anteil der Facharztqualifikation der Befragten unterscheidet sich teilweise deutlich zwischen im Krankenhaus tätigen Ärzten und Kollegen in der Niederlassung. Insbesondere ist der Anteil der Facharztqualifikation in Kinderchirurgie im Niedergelassenenbereich deutlich niedriger mit 7,5 % zu 23 % im Krankenhaus, Tab. 2. In der weiteren Darstellung wird die Weiterbildungskategorie Unfallchirurgie nicht mehr ausgewiesen, da diese in die Kategorie spezielle Unfallchirurgie komplett integriert ist.

**Table 1:** 

Facharztqualifikation *n* = 772	CA (%)	OA (%)	ASS (%)	NL (%)
Chirurgie	62,71	33,67	4,24	44,63
Spezielle Unfallchirurgie	62,15	37,67	2,54	32,20
Orthopädie und Unfallchirurgie	70,62	67,00	66,10	78,56

Position *CA* Chefärzte, *OA* Oberärzte, *ASS* Assistenten, *NL* Niedergelassene

**Table 2:** 

Facharztqualifikation	Krankenhaus (%, *n* = 578)	Niederlassung (%, *n* = 160)
Chirurgie	35,81	43,13
Spezielle Unfallchirurgie	38,41	22,75
Orthopädie und Unfallchirurgie	67,47	78,13
Kinderchirurgie	22,84	7,50

### Versorgungshäufigkeit von Unfallpatienten im Kindes- und Jugendalter

Die Versorgungsfrequenz ist von Frakturen im Kinder- und Jugendalter ist unterschiedlich.

**Figure 1:**
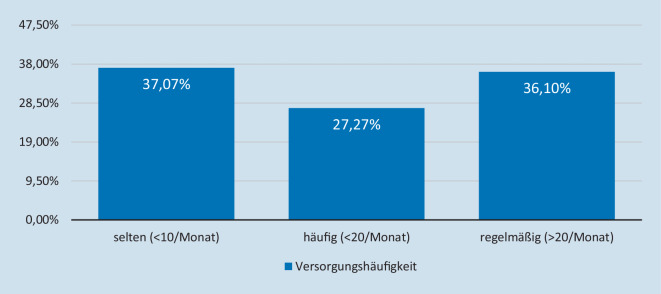

Aus den Antworten von 759 Teilnehmern zeigt sich, dass über ein Drittel der Befragten weniger als 10 Frakturen im Monat versorgen, Abb. 1.

Weniger als 10 Kontakt pro Monat ergaben sich bei 37 %, 27 % hatten unter 20/Monat und 36 % regelmäßig mit mehr als 20 Frakturen im Monat Kontakt, Tab. 3. Je nach Facharztqualifikation variiert auch die Frequenz der Behandlungen dieser Patienten. Kinderchirurgen behandeln in mehr als 80 % mehr als 20 kindliche und jugendliche Patienten mit Frakturen im Monat, Tab. 3.

**Table 3:** 

Frequenz	Gesamt	CH	KiCH	O + U	SUCH
Anzahl	759	289	143	520	283
Selten (< 10/Monat, %)	37,07	30,10	7,69	43,25	31,41
Häufig (< 20/Monat, %)	27,27	35,29	11,19	30,14	34,66
Regelmäßig (> 20/Monat, %)	36,10	34,60	81,12	27,20	33,94

Facharztqualifikation *CH* Chirurgie, *KiCH* Kinderchirurgie, *O* *+* *U* Orthopädie und Unfallchirurgie, *SUCH* spezielle Unfallchirurgie

## Frakturdiagnostik

### Primäre Röntgendiagnostik in zwei Ebenen

Befragt nach der Forderung nach einer exakten zweiten Röntgenebene auch bei starker Dislokation der Fragmente geben von der Gesamtheit der Befragten 52 % an, diese standardmäßig zu fordern. Die Kinderchirurgen unterscheiden sich mit 25 % deutlich von den anderen Facharztgruppen. Die Gruppe der Niedergelassenen besteht in 69 % auf einer zweiten Ebene (Tab. 4).

**Table 4:** 

	Gesamt	CH	KiCH	O + U	SUCH	CA	OA	ASS	NL
Anzahl	733	282	140	502	275	165	291	125	152
Standardmäßig immer (%)	51,84	48,23	25,00	57,77	51,64	42,42	43,64	62,40	69,08
Eine Ebene reicht mir (%)	2,05	2,13	71,00	2,39	1,09	1,21	1,72	1,60	3,95
Bei stark dislozierten Frakturen reicht mir eine Ebene (%)	46,11	49,65	74,29	39,84	47,27	56,36	54,64	36,00	26,97

Facharztqualifikation *CH* Chirurgie, *KiCH* Kinderchirurgie, *O* *+* *U* Orthopädie und Unfallchirurgie, *SUCH* spezielle Unfallchirurgie; Position *CA* Chefärzte, *OA* Oberärzte, *ASS* Assistenten; *NL* Niedergelassene

### Röntgen der Gegenseite

Bei der Frage, ob nach einem Unfallereignis bei unklaren Befunden in der Röntgendiagnostik die Gegenseite bei Kindern und Jugendlichen geröntgt wird, geben von 759 aller Befragten 69,57 % an, dies nie zu veranlassen, Tab. 5. 27,93 % benutzen dies selten, 2,11 % häufig und 0,40 % als Standard. Vergleicht man den Status der Weiterbildung, so zeigt sich, dass die Kinderchirurgen in 87,41 % die Indikation zum Röntgen der Gegenseite ablehnen.

**Table 5:** 

	Gesamt	CH	KiCH	O + U	SUCH	CA	OA	ASS	NL
Anzahl	759	289	143	520	277	171	295	127	166
Nie (%)	69,57	68,51	87,41	65,19	68,95	67,84	73,56	66,93	66,27
Selten (%)	27,93	29,76	12,59	31,15	29,96	28,65	25,08	31,50	29,52
Häufig (%)	2,11	1,04	0,00	3,08	0,72	2,34	1,36	0,79	4,22
Immer (%)	0,40	0,69	0,00	0,58	0,36	1,17	0,00	0,79	0,00

Facharztqualifikation *CH* Chirurgie, *KiCH* Kinderchirurgie, *O* *+* *U* Orthopädie und Unfallchirurgie, *SUCH* spezielle Unfallchirurgie; Position *CA* Chefärzte, *OA* Oberärzte, *ASS* Assistenten; *NL* Niedergelassene

### Frakturdiagnostik mit Ultraschall

Der Ultraschall wird in der Frakturdiagnostik in der gesamten Ärztegruppe in 23 % verwendet, in 40 % kommt er gelegentlich zum Einsatz. Die niedergelassenen Kolleginnen und Kollegen nutzen die Ultraschallfrakturdiagnostik am häufigsten mit 39 %, und weitere 32 % nutzen sie gelegentlich, Tab. 6.

**Table 6:** 

	Gesamt	CH	KiCH	O + U	SUCH	CA	OA	ASS	NL
Anzahl	733	282	140	502	275	165	291	125	152
Ja (%)	23,19	23,05	29,29	21,91	18,91	18,79	18,21	20,80	39,47
Gelegentlich (%)	40,25	36,88	51,43	39,24	38,18	43,03	41,58	43,20	32,24
Nur zur Verlaufskontrolle (%)	5,05	5,32	3,57	4,98	6,18	8,48	5,50	1,60	3,29
Nein (%)	31,51	34,75	15,71	33,86	36,73	29,70	34,71	34,40	25,00

Facharztqualifikation *CH* Chirurgie, *KiCH* Kinderchirurgie, *O* *+* *U* Orthopädie und Unfallchirurgie, *SUCH* spezielle Unfallchirurgie; Position *CA* Chefärzte, *OA* Oberärzte, *ASS* Assistenten; *NL* Niedergelassene

### Röntgen bei polytraumatisierten Kindern und Jugendlichen

Bei dieser Frage wurde der Einsatz der Röntgendiagnostik auf den Einsatz des Ganzkörper-CT bei polytraumatisierten Kindern und Jugendlichen beschränkt. Nach der Verwendung spezieller CT-Kinder-Protokolle wurde nicht gefragt.

18 % aller Befragten sehen nie, 50 % selten die Indikation zum Trauma-Scan in dieser Altersgruppe. Standardmäßig werden von 14 % aller Befragten Ganzkörper-CT bei polytraumatisierten Kindern unter 12 Jahren angeordnet. Niedergelassene wurde bei der Auswertung nicht berücksichtigt. Kinderchirurgen zeigen sich mit 6,5 % Anteil beim standardmäßigen Einsatz des Trauma-Scans zurückhaltender als andere Facharztgruppen (Tab. 7).

**Table 7:** 

	Gesamt	CH	KiCH	O + U	SUCH	CA	OA	ASS
Anzahl	725	279	139	497	273	164	290	124
Nie (%)	18,07	18,64	15,83	17,91	13,55	11,59	11,03	11,29
Selten (%)	50,34	50,54	61,87	46,88	49,82	55,49	54,83	56,45
Häufig (%)	17,66	16,85	15,83	18,51	19,78	17,07	20,34	15,32
Standardmäßig (%)	13,93	13,98	6,47	16,70	16,85	15,85	13,79	16,94

Facharztqualifikation *CH* Chirurgie, *KiCH* Kinderchirurgie, *O* *+* *U* Orthopädie und Unfallchirurgie, *SUCH* spezielle Unfallchirurgie; Position *CA* Chefärzte, *OA* Oberärzte, *ASS* Assistenten

Der Anteil an den Gesamtbefragten, die standardmäßig das Ganzkörper-CT bei jungen polytraumatisierten Patienten älter als 12 Jahre einsetzen, steigt im Vergleich zur jüngeren Gruppe um 5,5 %. Kollegen mit der Facharztqualifikation spezielle Unfallchirurgie sehen eine vermehrte Indikation zum Einsatz des Polytrauma-CT bei der älteren Gruppe um 8 %, Tab. 8.

**Table 8:** 

	Gesamt	CH	KiCH	O + U	SUCH	CA	OA	ASS
Anzahl	725	279	139	497	273	164	290	124
Nie (%)	14,07	15,05	10,07	13,48	10,26	7,93	7,59	8,87
Selten (%)	36,97	34,41	54,68	32,60	31,50	38,41	41,03	42,74
Häufig (%)	29,38	27,96	27,34	30,38	33,33	32,32	30,34	29,03
Standardmäßig (%)	19,59	22,58	7,91	23,54	24,91	21,34	21,03	19,35

Facharztqualifikation *CH* Chirurgie, *KiCH* Kinderchirurgie, *O* *+* *U* Orthopädie und Unfallchirurgie, *SUCH* spezielle Unfallchirurgie; Position *CA* Chefärzte, *OA* Oberärzte, *ASS* Assistenten

## Diskussion

Die Röntgendiagnostik bei Frakturen im Kindes- und Jugendalter ist etablierter Standard. Die Vorteile und Erfolge dieser Diagnostik sind anerkannt, es bedarf aber auch heute immer einer rechtfertigenden Indikation auch zur Röntgendiagnostik. Es sollten Anamnese und klinische Untersuchung der Fraktur oder Verletzung immer *vor* der Indikation zur Röntgendiagnostik erfolgen, um diese zu erhalten. Bereits 1992 formulierte der Radiologe Alzen, dass angesichts der großen Diskrepanz zwischen klinischem Verdacht und radiologischer Bestätigung von Frakturen Röntgenuntersuchungen bei Bagatellverletzungen im Kindesalter nur bei strenger Indikation durchgeführt werden sollten. Obwohl der Arzt gesetzlich zu einer ausführlichen Dokumentation jedes Falles verpflichtet ist, bedeute dies nicht zwangsläufig, dass er immer eine Röntgenaufnahme durchführen müsse [6]. Grundsätzlich ist die Anwendung von Röntgenstrahlung eine Körperverletzung, es sei denn, sie ist konform mit der Röntgenverordnung. Auch der Bundesgerichtshof weist darauf hin, dass nur medizinisch notwendige Röntgenuntersuchungen keine Körperverletzung sind, da durch den Einsatz der ionisierenden Strahlen Langzeitschäden auftreten könnten [61]. Der kindliche Organismus ist im Vergleich zum Erwachsenen strahlensensibler und damit anfälliger, durch ionisierende Strahlen Malignome zu entwickeln, auch weil Kinder und Jugendliche eine längere Lebenserwartung und damit Zeitspanne haben als Erwachsene [16, 42, 47, 49].

Bei Kindern muss besonders auf die jeweilige individuelle Abwägung zwischen Nutzen und Risiko beim Einsatz dieser Technik Wert gelegt werden. Dem ALARA-Prinzip sollte in dieser Altersgruppe ganz konsequent gefolgt werden [29, 35, 40, 42, 49, 62]. Bei der Indikation zur Röntgenuntersuchung zum Ausschluss einer knöchernen Verletzung sollten immer individuell Nutzen und Risiko abgewogen werden [62]. Medikolegaler und teilweise der Druck der Erziehungsberechtigten auf die behandelnden Kindertraumatologen, insbesondere bei Nachkontrollen Röntgenaufnahmen zu veranlassen, sollten in den Hintergrund gestellt werden. Insbesondere, wenn diese vom Behandler als nichtindiziert eingestuft werden [19]. Häufig ist den Erziehungsberechtigten die Wirkung ionisierender Strahlung auf den jungen Organismus nicht geläufig [41, 52]. Aber es muss auch das Wissen über Strahlenvermeidung auf der Behandlerseite vertieft werden [11, 14, 43]. Die Strahlendosis wird häufig zu niedrig eingeschätzt [50, 52].

Bei den heutigen digitalen Röntgenbildern soll, ehe eine Aufnahme wiederholt wird, das Potenzial der digitalen Technik voll ausgereizt werden, eine optimale diagnostischer Aussagekraft zu erreichen, bevor wie früher ein neues Röntgenbild mit besserer Einstellung geschossen wird. Ein ungünstig belichtetes Röntgenbild ist keine Indikation, die Gegenseite zum Vergleich zu röntgen.

Röntgenaufnahmen des knöchernen Skeletts werden üblicherweise in 2 Ebenen angefertigt, da die Einzelaufnahme nur ein zweidimensionales Bild ermöglicht und damit die Beurteilung im Raum und Überlagerungen schwierig macht. 52 % der Befragten verlangen standardmäßig immer 2 Ebenen. Bei stark dislozierten Frakturen genügen 46 % der Befragten eine Ebene. Kinderchirurgen verlangen in über 70 % nur eine Ebene bei den Kindern und Jugendlichen mit Frakturverdacht. Die SKT erklärt hierzu, dass der fehlende Nachweis einer Fraktur in einer Röntgenebene nicht den Verzicht auf eine zweite rechtfertigt, denn es bestehe die Gefahr einer nur in einer Ebene dislozierten Fraktur [29].

Die SKT empfiehlt, dass bei klarer Operationsindikation die zweite Ebene dann in der Narkose zur Reposition oder Operation nachgeholt werden sollte [29]. Nur 2 % verzichten routinemäßig komplett auf die zweite Ebene. Kollegen in der Niederlassung verlangen in fast 70 % immer 2 Röntgenebenen und sind damit konform den Anforderungen der Bundesärztekammer. In der Leitlinie der Bundesärztekammer zur Qualitätssicherung in der Röntgendiagnostik wird für Skelett und Extremitäten die Darstellung in typischen Projektionen bei Standardlagerung mit angrenzendem Gelenk, in der Regel in 2 Ebenen, gefordert [20]. Dies wird für Kinder nicht modifiziert. Trotzdem erscheint das Verhalten vieler Befragter, dem Kind Schmerzen durch eine erzwungene zweite Ebene zu ersparen und unter der Narkose bei Reposition oder Operation die zweite Ebene zu dokumentieren, eindeutig kindgerechter. So sieht es auch der Konsens der SKT [29].

Die Gegenseite wird bei unklaren Befunden von zwei Drittel der Gesamtbefragten nie, bei den Kinderchirurgen in 87 % nie geröntgt. Von der Gesamtheit nutzen nur 2,5 % häufig oder immer das Röntgen der Gegenseite bei unklaren Befunden. Sowohl nach dem ALARA-Prinzip als auch nach der Röntgenverordnung ist die Indikation zum Röntgen der unverletzten Gegenseite sehr kritisch bis obsolet einzuschätzen; es besteht keine rechtfertigende Indikation [45, 55]. Die kanadische Röntgengesellschaft sieht in ihren Guidelines ebenfalls keine Indikation zum Röntgen der Gegenseite bei Kindern mit einem Empfehlungsgrad B [21]. Die Strahlenschutzkommission stuft das Röntgen der Gegenseite ebenso wie die Leitlinie als obsolet ein [34, 58]. Die Konsensempfehlung der SKT stützt diese Aussage [29].

Zahlreiche Arbeiten belegen den sinnvollen Einsatz der Sonographie in der Akuttraumatologie bei Kindern und Jugendlichen [2, 27, 32, 51]. Sowohl lange Röhrenknochen, hier besonders der proximale Humerus und der Ellenbogen [10, 22, 44], speziell auch der Unterarm und distaler Radius [8, 36] und Frakturen der Klavikula [27] eignen sich gut für den sonographischen Frakturnachweis. Auch in den USA wird in den letzten Jahren mehr und mehr in der Notfallbehandlung die Sonographie als „point-of-care ultrasonography“ (POCUS) eingesetzt [3]. Auch berichten zahlreiche Autoren über die validen sonographischen Kontrollen nach Repositionen der langen Röhrenknochen [9, 24, 31, 57, 60]. Die SKT spricht sich eindeutig in ihrem Konsenspapier für den vermehrten Einsatz der Sonographie in der Kindertraumatologie aus [29]. Die Ultraschalldiagnostik wird von über 30 % aller Befragten gar nicht eingesetzt, 40 % nutzen diese Technik gelegentlich. Von den Kinderchirurgen setzen 29 % die Sonographie bei Frakturen regelhaft, in 37 % gelegentlich ein.

Bei Kindern und Jugendlichen wird das Ganz-Körper-CT vor dem 12. Lebensjahr in 31 % häufig oder standardmäßig, über 12 Jahren zu fast 50 % eingesetzt.

Der unmittelbare Nutzen und Vorteil im Notfall kann immens sein, aber es liegen auch Daten vor, die das spätere Krebsrisiko hervorheben [1, 5, 15, 23]. Es steht außer Frage, dass das CT eingesetzt wird, wenn bei instabilen Kreislaufverhältnissen keine Klärung mit Thoraxröntgen und Sonographie kurzfristig erzielt werden kann [54]. Nach der S3-Polytrauma-Leitlinie sollte bei schwer verletzten Kindern eine zeitnahe Ganzkörper-Computertomographie mit traumspezifischem Protokoll erfolgen, auch wenn der Nachweis zur Reduzierung der Mortalität durch eine Ganzkörper-CT bei Kindern noch ausstehe [28].

Im Rahmen des Primary Survey ist sowohl leitlinienkonform als auch ATLS-konform die Sonographie als E-FAST etabliert [28, 30, 48, 54]. In der S3-Polytrauma-Leitlinie wird darauf hingewiesen, dass ein negatives Ergebnis der Basisuntersuchung mit E-FAST bei Kindern eine negative intraabdominelle Verletzung keineswegs ausschließe und eine Überwachung, ggf. ausführliche Wiederholungsuntersuchung oder eine CT-Untersuchung durchgeführt werden solle [28].

Brenner weist darauf hin, dass bei CT-Untersuchungen von Kindern diese eine höhere Organdosis als Erwachsene erhalten, und dass zahlreiche kindliche Organe sensibler für strahleninduzierte Krebserkrankungen sind [15, 17, 46]. Hier sollten altersgerechte Einstellungsprotokolle der Computertomographen verwendet werden, um die geringste sinnvolle Dosis einzusetzen [29, 38, 48, 56]. Es sollten diese Niedrigdosen-Pädiatrie-Protokolle und andere Techniken wie Flash-CT, Reduzierung der Dünnschnitt-CT-Bildgebung Anwendung finden [4, 7, 12, 13, 25, 53, 54]. In Kinderzentren wird allgemein durch vermehrte Nutzung spezieller Kinderprotokolle bei CT-Untersuchungen eine niedrigere Strahlendosis erzielt als in allgemeinen Erwachsenen-Traumazentren [18, 26]. Algorithmen können bei schwer verletzten Kindern ein Weg sein, die Indikation zum CT einzugrenzen und damit Strahlung einzusparen [37].

## Fazit

Bei der Umfrage über den aktuellen Stand beim Einsatz bildgebender Verfahren im Kindes- und Jugendalter ergibt sich insgesamt ein heterogenes Bild. Die Möglichkeiten der Strahlenreduktion in der Akutdiagnostik scheinen nicht komplett durchgängig genutzt zu werden, z. B. durch Verzicht auf Röntgen der Gegenseite bei unklaren Befunden oder durch Einsatz des Ultraschalls *bei einigen Frakturlokalisationen*. Die Umfrage zeigt: Nach einem Unfall wird die Ultraschalldiagnostik nur relativ selten eingesetzt.

Es bleibt weiterhin die Aufgabe für alle, die in der Kindertraumatologie tätig sind, zu sensibilisieren, um den Strahlenschutz für unsere jungen Patienten zu optimieren. Das ALARA-Prinzip muss hohe Priorität in der Diagnostik von Kindern und Jugendlichen haben.

## Footnotes

1 Aus Gründen der besseren Lesbarkeit und Verständlichkeit des Textes wird hier das generische Maskulinum als geschlechtsneutrale Form verwendet. Diese Form impliziert immer alle Geschlechter.
